# Assessment of the Microbiological Quality of Raw Milk Sold Through Vending Machines at the Farm Level in Switzerland

**DOI:** 10.3390/pathogens15030322

**Published:** 2026-03-17

**Authors:** Thomas Paravicini, Marc J. A. Stevens, Karen Barmettler, Nicole Cernela, Roger Stephan

**Affiliations:** Institute for Food Safety and Hygiene, Vetsuisse Faculty, University of Zurich, 8057 Zurich, Switzerland

**Keywords:** raw milk, food safety, foodborne pathogens, *Campylobacter* spp., STEC, *S. aureus*, *Y. enterocolitica*, *L. monocytogenes*, ESBL, MRSA

## Abstract

The sale of raw milk via vending machines represents a well-established distribution model in many European countries, including Switzerland. As part of this study, data on the microbiological quality of raw milk sold via vending machines in Switzerland were collected. A total of 124 raw milk samples from 124 raw milk vending machines across Switzerland were analysed. In addition to standard hygiene parameters (TVC and *E. coli*), the scope of the investigation particularly included foodborne pathogens as well as methicillin-resistant *Staphylococcus aureus* (MRSA) and extended-spectrum β-lactamase (ESBL)-producing Enterobacterales. Isolates were further characterised by whole-genome sequencing. Shiga toxin-producing *Escherichia coli* (STEC) were detected in 3.2%, *Staphylococcus aureus* was detected in 12.1%, *Listeria monocytogenes* was detected in 2.4%, *Campylobacter* spp. were detected in 1.6%, *Yersinia enterocolitica* was detected in 29.8%, and *Salmonella* spp. were detected in 0% of the samples. MRSA and ESBL-producing Enterobacterales were each detected in 0.8% of samples. The results highlight the potential risk of foodborne infections associated with the consumption of untreated raw milk, as well as hygiene deficiencies linked to several raw milk vending machines. Based on the generated data, the importance of the requested heat treatment of raw milk in Switzerland is clearly underscored. Furthermore, more precise and binding guidelines for self-monitoring and the management of raw milk vending machines appear necessary.

## 1. Introduction

In Switzerland, raw milk at the bulk tank level is subject to strict and regular hygiene and quality controls as part of official milk testing [1]. This includes twice-monthly surveys of the total bacterial count (indicator of milking hygiene; requirement ≤ 80,000 CFU/mL), the somatic cell count (indicator of udder health; requirement ≤ 350,000 cells/mL), and the detection of inhibitors (requirement: negative test). Despite this comprehensive monitoring, raw milk is not necessarily free of potentially pathogenic bacteria [2,3,4,5,6]. These can enter the milk through subclinical mastitis or contamination during the milking process [7,8,9]. If raw milk is consumed untreated, however, there is a direct potential for exposure to pathogens [9,10].

Internationally, the consumption of raw milk has increased in recent years, accompanied by a rise in reported outbreaks, highlighting the need for robust monitoring systems [11]. In particular, vulnerable population groups like young children, pregnant women, and immunocompromised individuals are at greatest risk, as infections can lead to severe outcomes [12].

In order to protect consumers, raw milk may not be advertised or offered for direct consumption in Switzerland [13]. However, it may be sold directly from the farm, provided that it is clearly labelled as raw milk and accompanied by appropriate processing instructions (heating to over 70 °C, storage below 5 °C, and consumption within 3 days are required) [13]. Such farm-gate sales typically take place via self-service milk vending machines, which enable consumers to purchase untreated raw milk directly. Thus, the responsibility for correct handling lies entirely with the consumer.

Findings from outbreak investigations [9] and surveys of users of such milk vending machines [14,15] show that the recommended heating of raw milk is not always carried out as recommended. This behaviour substantially increases health risk, as consumption of unpasteurised dairy products is associated with an 840-fold higher likelihood of illness and a 45-fold higher likelihood of hospitalisation compared with pasteurised products [16]. Nevertheless, some people prefer raw milk, whether due to taste preferences, regional identity, a direct relationship with the farm, or perceived health aspects [17,18]. In particular, potential protective effects in the context of allergies and asthma, as well as a slightly better availability of micronutrients (e.g., whey proteins, lactoferrin [19], and alkaline phosphatase [20]) compared to pasteurised milk, have been described in the literature [17,18,21,22,23]. Such factors contribute to raw milk being perceived by some consumers as “more natural”, higher quality, or unprocessed. However, scientific evidence for these positive effects often remains limited, as many reported associations arise from observational studies and are strongly influenced by environmental and lifestyle factors [8].

In addition to risks inherent to raw milk itself, milk vending machines present a further microbiological challenge, as suboptimal cooling, inconsistent cleaning intervals, and the potential formation of biofilms can facilitate bacterial growth or recontamination [9,15,24,25,26].

Despite these uncertainties, raw milk continues to be sold in Switzerland. In 2024, around 4700 tonnes of raw milk were produced in Switzerland for open sale, including milk vending machines (1.2% of the drinking milk produced). This corresponds to a calculated per capita production of 0.5 kg per person/year [27].

If diseases and outbreaks linked to raw milk occur, they are most often caused by *Campylobacter* spp., Shiga toxin-producing *Escherichia coli* (STEC), or *Salmonella* spp. [9]. These pathogens typically enter the milk via faecal contamination during or after milking (poor hygiene). Additional bacterial hazards such as *Listeria* (*L.*) *monocytogenes*, *Yersinia* (*Y.*) *enterocolitica*, and enterotoxin-producing *Staphylococcus* (*S.*) *aureus*, as well as viral agents including TBE virus or HPAI viruses, may also cause illness following the consumption of raw milk [9,10,28,29,30,31,32]. As no dedicated monitoring system exists for raw milk sold directly to consumers in Switzerland, assessing the occurrence of these pathogens remains essential for evaluating potential public health risks.

To date, only a single study has investigated the quality and safety of raw milk from vending machines in Switzerland [26]. In that study, 61 raw milk vending machines in northern and Central Switzerland were sampled. Although the authors reported the need for improved hygiene, no major pathogens such as *Campylobacter* spp., STEC, or *L. monocytogenes* were detected. Nevertheless, studies from neighbouring countries demonstrate that pathogens, including *Campylobacter* spp., *Salmonella* spp., *L. monocytogenes*, STEC, and *S. aureus,* may occur in raw milk at low prevalence and can lead to cases of illness [10,33,34,35,36,37,38,39]. In addition, data on antimicrobial-resistant bacteria and on the prevalence of pathogens such as *Y. enterocolitica* in raw milk remain scarce, and genomic characterisation of isolates from raw milk vending machines has rarely been performed. The present study, therefore, aimed to provide a more comprehensive assessment of the microbiological quality of raw milk from milk vending machines in Switzerland, using a larger and geographically more diverse sample set. In addition, isolates were further characterised using whole-genome sequencing, enabling deeper insight into genomic features and epidemiology.

## 2. Materials and Methods

### 2.1. Sample Collection

Sample collection was performed between June and October 2025. The locations of raw milk vending machines were primarily identified using the “milk vending machine finder” provided by Swissmilk “www.swissmilk.ch (accessed on 2 June 2025)”. All the raw milk samples were collected aseptically by the same person following an identical standardised procedure. At each vending machine, one 500 mL sample of raw milk was collected into a sterile bottle. Sampling was done without prior notification of the farmers. Samples were transported under chilled conditions and stored at 4 °C in the laboratory until further analysis. All analyses were initiated within 24 h after sample collection.

At each vending machine, the displayed milk temperature (when available) was recorded, and compliance with mandatory labelling requirements regarding raw milk treatment was verified.

### 2.2. Total Viable Counts (TVCs)

Samples were examined for TVCs according to ISO 4833-1:2013 [40]. Serial decimal dilutions of the raw milk samples were prepared using 0.85% NaCl. The raw milk (0.1 mL) and the dilutions (0.1 mL) were streaked onto plate count agar plates (Oxoid, Pratteln, Switzerland) and incubated for 72 ± 3 h at 30 ± 1 °C under aerobic conditions. Colonies were enumerated, and results were expressed as colony-forming units per millilitre (CFU/mL) of raw milk. The detection limit of the method was 1 log CFU/mL. From samples with total viable counts > 8.0 × 10^4^ CFU/mL, representative colonies were identified using matrix-assisted laser desorption ionisation–time of flight mass spectrometry (MALDI-TOF-MS, Bruker Daltonics, Bremen, Germany) and the software Flex Control 3.4., the MALDI Biotyper (MBT), Compass database version 4.1.100, and the MBT Compass BDAL 12.0 Library were applied.

### 2.3. ß-Glucuronidase-Positive Escherichia coli (E. coli)

Samples were examined quantitatively for *ß*-Glucuronidase-positive *E. coli* according to ISO 16649-2:2001 with slight modifications [41]. In total, 0.1 mL of raw milk was streaked onto Rapid *E. coli* agar (Biorad Laboratories AG, Cressier, Switzerland) and incubated at 37 ± 1 °C for 18–24 h. Suspicious purple/blue colonies were counted and further identified using MALDI-TOF-MS analysis, applying the software Flex Control 3.4., the MALDI Biotyper (MBT), Compass database version 4.1.100, and the MBT Compass BDAL 12.0 Library. The detection limit of the method was 1 log CFU/mL. Due to its high genetic similarity, MALDI-TOF-MS does not reliably differentiate between *E. coli* and *Shigella* spp. Isolates identified as *E. coli* by MALDI-TOF-MS were reported as *E. coli*, since *Shigella* spp. are *ß*-Glucuronidase-negative.

### 2.4. Coagulase-Positive Staphylococcus spp. (Presumptive S. aureus)

Samples were examined quantitatively for coagulase-positive *Staphylococcus* spp. (presumptive *S. aureus*) according to ISO 6888-2:2021 [42]. In total, 0.1 mL of raw milk was streaked onto EASY Staph^®^ agar (Biokar Diagnostics, Allonne, France) and incubated at 37 ± 1 °C for 44 ± 4 h. Dark-to-light grey colonies showing a suspicious opaque halo were enumerated, and the most common morphotype was further identified by MALDI-TOF-MS analysis was performed applying the software Flex Control 3.4., the MALDI Biotyper (MBT) Compass database version 4.1.100, and the MBT Compass BDAL 12.0 Library. The detection limit of the method was 1 log CFU/mL.

### 2.5. Campylobacter spp.

Samples were examined quantitatively and qualitatively for *Campylobacter* spp. according to ISO 10272:2017 [43].

For quantitative detection, 0.1 mL of the raw milk sample was streaked onto Brilliance™ Campy count agar (Oxoid, Basingstoke, UK) as well as modified charcoal cefoperazone deoxycholate agar (m-CCDA; Oxoid, Basingstoke, UK). Plates were incubated under microaerophilic conditions for 44 ± 4 h at 41.5 °C. The detection limit of the method was 1 log CFU/mL.

For qualitative analysis, 10 mL of raw milk was enriched in 90 mL of Preston enrichment broth (Oxoid, Basingstoke, UK) and incubated under microaerophilic conditions at 41.5 °C overnight. The following day, the enriched samples were streaked onto Brilliance™ Campy count agar as well as m-CCDA plates and incubated under microaerophilic conditions at 41.5 °C for 44 ± 4 h. Suspicious dark-red colonies on Brilliance™ Campy count and/or greyish colonies on m-CCDA plates were further identified using MALDI-TOF-MS, applying the software Flex Control 3.4., the MALDI Biotyper (MBT) Compass database version 4.1.100, and the MBT Compass BDAL 12.0 Library.

### 2.6. Yersinia enterocolitica

Samples were examined quantitatively and qualitatively for *Y. enterocolitica* according to ISO 10273:2017 [44].

For quantitative detection, the raw milk (0.1 mL) and decimal dilutions in 0.8% sodium chloride (0.1 mL) were streaked onto Cefsulodin–Irgasan–Novobiocin Agar (CIN) (Oxoid, Basingstoke, UK) and CHROMagar© *Y. enterocolitica* (CHROMagar, Paris, France), which were incubated at 30 °C for 24 h. The detection limit of the method was 1 log CFU/mL.

For qualitative detection of *Y. enterocolitica*, enrichment in Peptone Sorbitol Bile (PSB) broth (Merck KGaA, Darmstadt, Germany) was performed. A total of 10 mL of raw milk was added to 90 mL of PSB broth. Prior to incubation, 10 mL of each PSB enrichment was transferred into 90 mL of Irgasan–Ticarcillin–Chlorate (ITC) broth (Merck KGaA, Darmstadt, Germany). Both enrichments were incubated at 25 °C for 44 h. Thereafter, to increase sensitivity for *Yersinia* spp. isolation, 0.5 mL of each enriched sample (PSB or ITC) was treated with 4.5 mL of 0.5% potassium hydroxide (KOH; Honeywell Fluka Fisher Scientific, Reinach, Switzerland) for 20 ± 5 s. A loopful of the suspension was then inoculated onto CIN and CHROM agar plates, which were incubated at 30 °C for 24 h. Potentially positive colonies, showing metallic blue (apathogenic) or mauve (pathogenic) colour on CHROMagar as well as pink colonies on CIN agar, were further identified using MALDI-TOF-MS by applying the software Flex Control 3.4., the MALDI Biotyper (MBT) Compass database version 4.1.100, and the MBT Compass BDAL 12.0 Library.

### 2.7. Listeria monocytogenes

Samples were examined qualitatively for *L. monocytogenes* according to ISO 11290-1:2017 [45]. A total of 10 mL of raw milk was enriched in 90 mL of Half–Fraser standard broth (Biorad Laboratories AG, Cressier, Switzerland) and incubated at 30 °C overnight. In a second enrichment step, 0.1 mL of the Half–Fraser broth sample was transferred into 10 mL of Fraser broth (BioRad Laboratories AG, Cressier, Switzerland) and incubated for another 24 ± 2 h at 37 °C. The next day, a loopful of the enriched suspension was inoculated onto Agar Listeria (AL) according to Ottaviani and Agosti (BioRad Laboratories AG, Cressier, Switzerland) and incubated at 37 °C for 48 ± 2 h. Suspicious blue colonies with a surrounding opaque halo were identified by MALDI-TOF-MS, applying the software Flex Control 3.4., the MALDI Biotyper (MBT) Compass database version 4.1.100, and the MBT Compass BDAL 12.0 Library.

### 2.8. Salmonella spp.

Samples were examined qualitatively for *Salmonella* spp. according to ISO 6579-1:2017 [46], with slight modifications. A total of 10 mL of the raw milk sample was enriched in 90 mL of buffered peptone water (Bio-Rad Laboratories AG, Cressier, Switzerland) and incubated at 37 °C overnight. The next day, 0.1 mL was transferred into 10 mL of Rappaport–Vassiliadis (RV) broth (Biorad Laboratories AG, Cressier, Switzerland) and 1 mL was transferred into 10 mL of Mueller–Kauffmann Tetrathionate Novobiocin broth (Biorad Laboratories AG, Cressier, Switzerland). RV broth was then incubated at 41.5 °C, and Mueller–Kauffman Tetrathionate Novobiocin broth was incubated at 37 °C for 24 ± 3 h. After incubation, the suspensions were streaked onto Xylose–Lysin–Deoxycholate (XLD) agar (BioRad Laboratories AG, Cressier, Switzerland) and Rapid Salmonella agar (RSAL, BioRad Laboratories AG, Cressier, Switzerland) and incubated at 37 °C for 24 ± 3 h. Pink colonies with a black centre on XLD agar and magenta colonies on RSAL agar were further analysed using MALDI-TOF-MS by applying the software Flex Control 3.4., the MALDI Biotyper (MBT) Compass database version 4.1.100, and the MBT Compass BDAL 12.0 Library.

### 2.9. Screening for STEC

Samples were examined qualitatively for *stx1/stx2* using real-time PCR. Ten millilitres of raw milk was enriched at a 1:10 ratio in Enterobacteriaceae enrichment (EE) broth (Becton, Dickinson, Heidelberg, Germany) for 24 h at 37 °C. One loopful of each of the enrichment cultures was then streaked onto sheep blood agar (Difco™ Columbia Blood Agar Base EH; Becton Dickinson AG, Allschwil, Switzerland) using the streak-plate method. The resulting colonies were suspended in 2 mL 0.85% NaCl. Samples were then screened by real-time PCR for *stx1* and *stx2* using the Assurance GDS^®^ for Shiga Toxin Genes (BioControl Systems, Bellevue, WA, USA).

### 2.10. Screening for Methicillin-Resistant Staphylococcus aureus (MRSA)

Samples were qualitatively examined for MRSA. For this purpose, a two-step enrichment procedure was conducted, first in Mueller–Hinton broth (MHB, Thermo Fisher Scientific, Waltham, MA, USA) supplemented with 6.5% NaCl (10 mL, 24 h at 37 °C) and afterwards in tryptic soy broth (TSB) supplemented with 75 mg/L aztreonam and 5 mg/L cefoxitin (24 h at 37 °C). After enrichment, samples were streaked onto Oxoid Brilliance MRSA Agar (Oxoid Ltd., Hampshire, UK) and incubated for 24 h at 37 °C. Presumptive positive colonies were further identified using MALDI-TOF-MS, applying the software Flex Control 3.4., the MALDI Biotyper (MBT) Compass database version 4.1.100, and the MBT Compass BDAL 12.0 Library.

### 2.11. Screening for ESBL-Producing Enterobacterales

Samples were examined qualitatively for ESBL-producing Enterobacterales. A total of 10 mL of raw milk was enriched in 90 mL of Enterobacteriaceae enrichment (EE) broth (BD, Franklin Lakes, NJ, USA) at 37 °C for 24 h. One loopful of each of the EE cultures was then streaked onto Brilliance ESBL^TM^ agar (Oxoid, Hampshire, UK) and incubated at 37 °C for 24 h. According to the chromogenic characteristics specified in the manufacturer’s guidelines, *E. coli* produces blue or pink colonies. In contrast, species belonging to the Klebsiella–Enterobacter–Serratia–Citrobacter (KESC) group appear as green colonies, and organisms of the *Proteus–Morganella–Providencia* (PMP) group display tan colonies with brown halos. If plates contained colonies of different coloration, one colony of each colour was selected for further analysis. If plates contained multiple isolates of identical coloration, only one of the isolates was selected for further analyses. Colonies were identified using MALDI-TOF-MS, applying the software Flex Control 3.4., the MALDI Biotyper (MBT) Compass database version 4.1.100, and the MBT Compass BDAL 12.0 Library.

### 2.12. DNA Extraction and Whole-Genome-Sequencing (WGS)

Whole-genome sequencing was performed for isolates belonging to *S. aureus*, *Campylobacter* spp., *Y. enterocolitica*, *L. monocytogenes*, methicillin-resistant *S*. *aureus*, and ESBL-producing Enterobacterales. Prior to sequencing, isolates were cultivated on sheep blood agar plates (Columbia Base Agar, Bio-Rad Laboratories AG, Cressier, Switzerland; sheep blood defibrinated, Oxoid, Basingstoke, UK) to obtain axenic cultures with sufficient biomass for DNA extraction. Isolates were incubated under species-specific optimal growth conditions, including temperature and atmospheric requirements.

DNA was extracted using the DNeasy Blood & Tissue Kit (Qiagen, Hombrechtikon, Switzerland), and DNA libraries were produced with the Illumina DNA Prep. Tagmentation kit (Illumina, San Diego, CA, USA). Sequencing was performed on an Illumina MiniSeq sequencer (Illumina, San Diego, CA, USA). Genomes were assembled using the Skesa v2.5.1-based software shovill 1.1.1 [47,48,49] with default settings and a minimal contig length of 500 bp.

Species identification was done based on an average nucleotide identity (ANI) with a threshold of >94% using fastANI v1.33 with standard settings [50]. Genomes were compared to representative genomes of genera downloaded from NCBI in June 2023 (*S. aureus*), October 2024 (*Yersinia* spp., *Listeria* spp.), March 2025 (*Campylobacter* spp.), and January 2026 (*E. coli*) [51]. *S. aureus* virulence genes, including toxins, were identified by comparing them to the virulence factor database VFDB [52]. The comparison was performed in Ridom Seqsphere+ v10.0.6 with default settings. Antibiotic resistance in MRSA was determined using the amrfinderplus database [53]. Analyses were performed in Ridom Seqsphere+ v10.0.6 with default settings. The AMR patterns of ESBL *E. coli* were determined using a Resistance Gene Identifier (RGI) 6.0.3 and database version 3.2.7 [54]. Multilocus sequence typing (MLST) and core-genome MLST (cgMLST) were performed in Ridom Seqsphere+ v10.0.6 (Ridom GmbH, Münster, Germany) using the cgMLST-defined schemes for *L. monocytogenes* [55], *S. aureus* [56,57], and *Campylobacter* spp. [58]; an ad hoc scheme for *Y. enterocolitica* [59]; and the Warwick MLST scheme for (ESBL) *E. coli* [60,61]. Minimal spanning tree constructions were performed in Seqsphere+ using standard settings. For *Y. enterocolitica*, biotypes were determined by aligning core protein sequences, as described previously [59].

### 2.13. Descriptive Statistics

The confidence intervals (CIs) were calculated using the Wilson confidence interval.

## 3. Results

### 3.1. Sample Collection

A total of 124 raw milk samples were collected between June and October 2025. The samples represented in particular cantons with a larger number of farms operating vending machines. Distribution of the farms was as follows: Zürich (*n* = 29), Bern (*n* = 19), Aargau (*n* = 16), Thurgau (*n* = 14), Lucerne (*n* = 10), St. Gallen (*n* = 10), Basel-Landschaft (*n* = 8), Freiburg (*n* = 4), Schwyz (*n* = 4), Solothurn (*n* = 3), Zug (*n* = 3), Appenzell Ausserrhoden (*n* = 2), and Appenzell Innerrhoden (*n* = 2). The temperatures recorded at the milk vending machines ranged from 1.0 to 7.3 °C, with a median temperature of 3.5 °C. Two vending machines displayed temperatures above 6 °C. Ten vending machines had no temperature display, or the display did not work. Processing and handling instructions (storage below 5 °C, heating to 70 °C before consumption, and consumption within 3 days) were displayed in 119 out of 124 milk vending machines. One sample was obtained directly from the bulk tank by the farmer upon request.

### 3.2. Total Viable Counts (TVCs)

**Figure 1:**
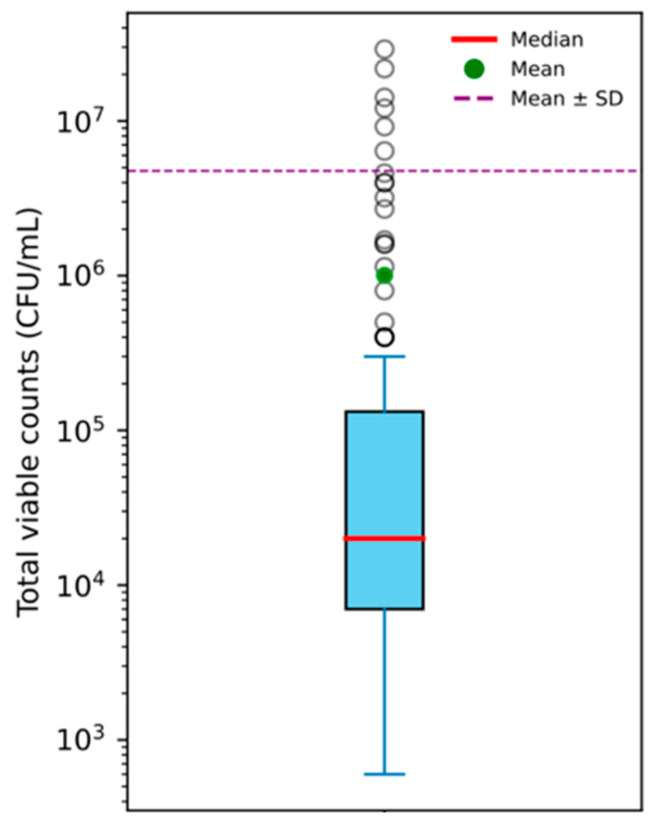
The distribution of the TVC values illustrated in a box plot.

Total viable counts (TVCs) ranged from 6 × 10^2^ CFU/mL up to 2.9 × 10^7^ CFU/mL, with a median value of 2 × 10^4^ CFU/mL (Figure 1).

The predominant bacterial species identified in the 37 samples (29.8%) exceeding a TVC of 8 × 10^4^ CFU/mL are listed in Table 1. Species identification using MALDI-TOF MS was successful in 33 out of 37 isolates (Table 1).

### 3.3. Escherichia coli (E. coli)

ß-Glucuronidase-positive *E. coli* was detected quantitatively in 34 out of 124 samples (27.42%, CI: 20.4–35.8%), with bacterial counts ranging from 10^1^ CFU/mL to 1.2 × 10^4^ CFU/mL. The median *E. coli* count was 5 × 10^1^ CFU/mL.

### 3.4. Coagulase-Positive Staphylococcus spp. (S. aureus)

*Staphylococcus aureus* was quantitatively detected in 15 out of 124 samples (12.1%, CI: 7.5–19.0%). *S. aureus* counts ranged from 10^1^ CFU/mL to 2.2 × 10^2^ CFU/mL (median: 3 × 10^1^ CFU/mL (Table 2). Sequence types and SE enterotoxin genes for the isolates are shown in Table 2. A total of eight different sequence types (STs) were identified. Only one ST504 isolate harboured SE enterotoxin genes.

**Figure 2:**
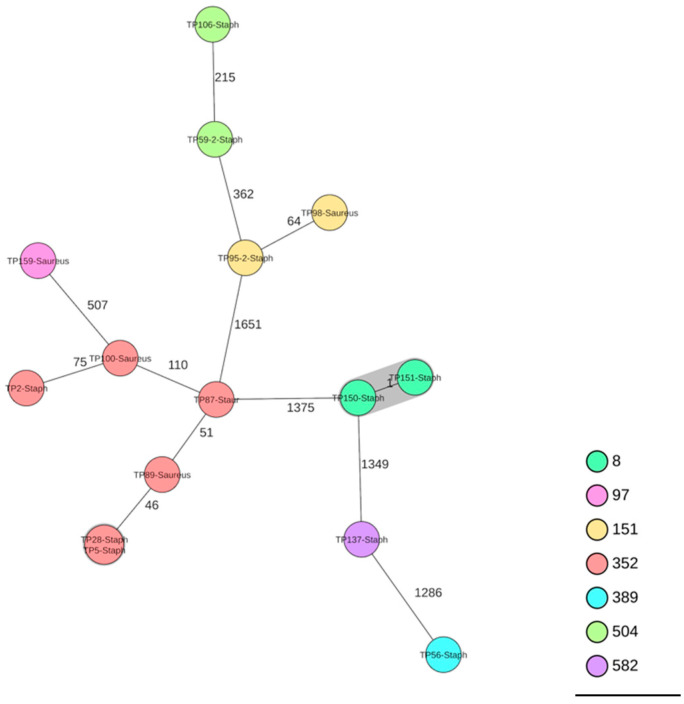
Minimum-spanning tree based on cgMLST allelic profiles of 15 *S. aureus* isolates. Each circle represents an allelic profile. The number on connecting lines represents the number of allelic differences between two strains. Each circle contains the strain ID(s). Grey shaded areas indicate clusters of isolates differing by fewer than 10 alleles (AD < 10).

A cgMLST-based analysis showed a high heterogeneity within the *S. aureus* isolates (Figure 2).

### 3.5. Campylobacter spp.

Two samples (TP 51 and TP 103) tested positive for *Campylobacter* spp. in the qualitative approach (1.6%, CI: 0.5–5.7%). Both isolates were identified as *Campylobacter jejuni*. Two different sequence types (STs) were identified: ST61 and ST not defined.

### 3.6. Yersinia enterocolitica

*Y. enterocolitica* was detected in 29.8% (CI: 22.5–38.4%) of the 124 samples. In total, 37 *Y. enterocolitica* isolates were detected, 17 (13.7%) of which had already been isolated in the quantitative approach (Table 3). The bacterial counts of the 17 samples ranged from 1.0 × 10^1^ CFU/mL to 1.9 × 10^6^ CFU/mL, with a median value of 1.3 × 10^3^ CFU/mL. All detected isolates belonged to biotype (BT) 1A, except for sample TP 111, for which no BT could be assigned. In total, four different STs were determined: ST3, ST8, ST118, and ST157.

**Figure 3:**
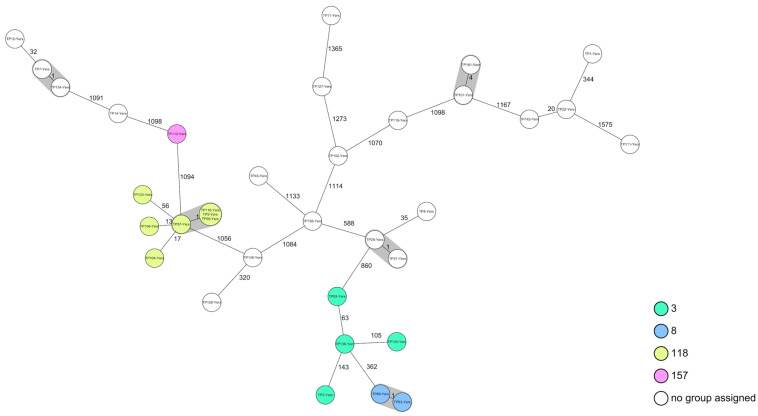
Minimum-spanning tree based on cgMLST allelic profiles of 35 *Y. enterocolitica* isolates. Each circle represents an allelic profile. The numbers on connecting lines represent the number of allelic differences between two strains. Each circle contains the strain ID(s). Grey shaded areas indicate clusters of isolates differing by fewer than 10 alleles (AD < 10).

A cgMLST-based analysis showed a high heterogeneity within the *Y. enterocolitica* isolates (Figure 3).

### 3.7. Listeria monocytogenes

*L. monocytogenes* was detected in 3 out of 124 samples (2.4%, CI: 0.8–6.9%). Three different sequence types (STs) were identified: ST37, ST226, and ST451.

### 3.8. Salmonella

None of the 124 raw milk samples tested positive for *Salmonella* (0%, CI: 0–3.0%).

### 3.9. Shiga-Toxin-Producing E. coli (STEC)

Four (3.2%, CI: 1.3–8.0%) of the 124 tested raw milk samples were positive for *stx* by PCR. Three samples were positive for *stx1*, and one sample was positive for *stx1* and *stx2*. The STEC strains could not be isolated in any of the four cases.

### 3.10. Methicillin-Resistant Staphylococcus aureus (MRSA)

A single milk sample among the 124 samples (0.8%, CI: 0.1–4.4%) tested positive for MRSA. The isolate (TP 124) was assigned to ST398 using MLST and carried the *mecA* gene.

### 3.11. ESBL-Producing Enterobacterales

A single milk sample among the 124 samples (0.8%, CI: 0.1–4.4%) tested positive for ESBL-producing Enterobacterales. The ESBL-producing *E. coli* (TP 21) isolate was assigned to ST16559 using the Achtman/Warwick MLST scheme. The isolate harboured the ESBL gene *bla*_CTX-M-14_.

## 4. Discussion

Raw milk has been repeatedly identified as a source of foodborne disease outbreak events in Europe in the past. According to risk assessments by the European Food Safety Authority (EFSA), *Campylobacter* spp., *Salmonella* spp., and STEC are considered the epidemiologically most relevant bacterial pathogens associated with raw milk [9]. The sale of raw milk via vending machines represents a well-established distribution model in many European countries, including Switzerland. However, numerous studies have shown that these vending machines may pose significant risks concerning microbiological safety [9]. In Switzerland, there is no obligation for operators to routinely self-check the microbiological quality of milk sold through vending machines. The present study aimed to comprehensively assess the microbiological quality of raw milk sold via vending machines. In total, 124 raw milk vending machines were sampled, predominantly located in the northern and eastern parts of Switzerland.

The aerobic mesophilic total viable counts (TVCs) of the samples showed a wide range, from 6 × 10^2^ CFU/mL to 2.9 × 10^7^ CFU/mL. Overall, 37 samples (29.8%) exceeded colony counts of 80,000 CFU/mL, which corresponds to the legal threshold for official milk testing applied to bulk tank milk twice monthly (VHyMP, 2005 [62]). In contrast, this threshold was exceeded in just 0.7% of the 372,020 bulk tank samples examined in milk testing of the year 2024 [63].

In total, 15 samples exhibited TVC values exceeding 1 × 10^6^ CFU/mL, including four samples with counts above 1 × 10^7^ CFU/mL. In these samples, the bacterial flora was primarily dominated by psychrotrophic *Pseudomonas* spp. The sporadically elevated TVC values, together with the marked deviation from the results of the official milk quality monitoring, indicate that, in a limited number of cases, relevant bacterial growth or contamination occurs at the level of raw milk vending machines. Such hygienic deficiencies associated with raw milk vending machines have also been reported in previous studies [15,26].

Whether the sporadically observed markedly elevated TVCs in the analysed raw milk samples can be attributed to prolonged storage of the milk within the vending machines (microbial proliferation within the milk) or to potential contamination originating from the vending machines themselves (e.g., due to biofilms) cannot be conclusively determined based on available data. However, due to their design, raw milk vending machines provide numerous potential niches for biofilm development, including valves, hoses, and storage containers [9,64]. Several bacterial genera isolated in the present study, including *Pseudomonas* spp., *Acinetobacter* spp., *Staphylococcus* spp., *Listeria* spp., and *E. coli*, have previously been detected in biofilms associated with dairy production and processing environments [9,65,66,67,68,69]. A study conducted in Slovenia investigated the hygienic status of raw milk vending machines and reported microbial contamination levels ranging from 1.8 log CFU/mL to 6.0 log CFU/mL on the internal surfaces of dispensing nozzles and collection containers [25].

*E. coli*, often used as an indicator organism for faecal contamination in raw milk, was detected in 34 samples (27.4% >10^1^ CFU/mL), with counts ranging from 10^1^ to 1.2 × 10^4^ CFU/mL. Growth of *E. coli* is not expected at the cooling temperatures (2–6 °C) targeted by raw milk vending machines [70,71]. The prevalence observed in the present study is largely consistent with the findings of a Swiss study from 2018 [26], which detected *E. coli* in 30.1% of samples with bacterial counts exceeding 10 CFU/mL.

*S. aureus* was detected in 15 samples (12.1%, >10 CFU/mL). Among the *S. aureus* isolates studied, only one strain (ST504, CC705) harboured genes encoding staphylococcal enterotoxins (*sec*, *sell*) and toxic shock syndrome toxin 1 (*tst1*), corresponding to 6.7% of all *S. aureus* isolates. Quantitative counts of *S. aureus* were low in all positive samples, with a maximum of 2.2 × 10^2^ CFU/mL. The enterotoxin gene-positive isolate was likewise detected at a low level of 10 CFU/mL, which is insufficient for enterotoxin production [72].

The prevalence of 12.1% observed in the present study is markedly lower than the 30.1% reported in an earlier Swiss study [26]. Meta-analyses have reported a mean prevalence of *S. aureus* in raw milk of 33.5% (95% CI: 29.5–37.7%) globally, and 22.8% (95% CI: 19.0–27.0%) within Europe [73]. Concurrently, studies reporting comparably low prevalence have been published, for example, from Northern Italy (*n* = 383; 9.1%) [74]. It should be taken into account that the observed prevalence of *S. aureus* at the bulk tank milk level may depend on herd size, particularly because *S. aureus* is regarded as a cow-associated mastitis pathogen. Direct comparisons of prevalence data with studies from other countries may, therefore, be of limited validity, as herd sizes in those settings are often substantially larger [75].

A notable finding of the present study was the low proportion of isolates encoding for enterotoxins (one isolate, 6.7%). By contrast, other studies have reported substantially higher proportions of enterotoxigenic *S. aureus* isolates in raw milk, including Switzerland (*n* = 22 isolates; 81.8%) [26], Northern Italy (*n* = 35 isolates; 45.7%) [74], and a global meta-analysis (39.3%) [73]. The enterotoxin gene profile identified in the present isolate (*sec*, *sell*) is consistent with previous reports indicating that *sec* is particularly prevalent among *S. aureus* isolates from milk and dairy products [73,76]. Multilocus sequence typing (MLST) of the *S. aureus* isolates (excluding the MRSA isolate) revealed the following sequence types: ST352 (*n* = 6; 40%), ST8 (*n* = 2; 13%), ST151 (*n* = 2; 13%), ST504 (*n* = 2; 13%), ST97 (*n* = 1; 7%), ST389 (*n* = 1; 7%), and ST582 (*n* = 1; 7%). At the clonal complex (CC) level, CC97 (*n* = 7; 47%) and CC705 (*n* = 4; 27%) were predominant, followed by CC8 (*n* = 2; 13%), CC15 (*n* = 1; 7%), and CC398 (*n* = 1; 7%). The most frequently identified sequence type was ST352 (CC97, six isolates). The predominance of CC97 and CC705 is consistent with previous studies [77,78] and reflects lineages typically associated with bovine mastitis [77,78,79,80,81,82,83]. Two isolates belonged to CC8, which is linked to genotype B, a lineage frequently associated with a high herd prevalence of mastitis [84] and previously described as a significant contaminant in Swiss raw milk cheeses [85].

*Campylobacter jejuni* was qualitatively detected in two (1.6%) raw milk samples. *Campylobacter* spp. are common commensals of the gastrointestinal tract of livestock animals, particularly in poultry [86]. However, they are also frequently detected in other food-producing animal species, such as cattle, as well as in companion animals and various environmental reservoirs [87,88,89]. In recent Swiss prevalence data regarding colonisation of the bovine gastrointestinal tract with *Campylobacter* spp., the pathogen was detected in 52% (*n* = 306) of caecal samples from calves, whereas a substantially lower prevalence of 10.2% (*n* = 935) was described for faecal samples from slaughter cattle [10,90].

To date, *Campylobacter* spp. have not been isolated from raw milk in Switzerland [26]. In Europe, meta-analyses have reported a pooled prevalence of *Campylobacter* spp. in raw milk of 1% (95% CI: 0–2%), which is in accordance with the findings of the present study [91].

*Campylobacter* spp. primarily enter the food chain through faecal contamination [92,93]. While the majority of infections are attributed to improper handling or processing of poultry meat, multiple outbreak investigations have also identified raw milk as a relevant source [94]. This is exemplified by a foodborne disease outbreak reported in Switzerland in 2024, in which the consumption of raw milk was associated with *Campylobacter jejuni* infections among children [10]. Based on available scientific evidence, the European Food Safety Authority (EFSA) considers *Campylobacter* spp. to be the most common cause of foodborne infections associated with consumption of raw milk [9].

In bovine faecal samples from Switzerland, sequence types ST21 (21%), ST61 (12%), and ST48 (11%) are among the most frequently detected [95]. Sequence type 61 is predominantly associated with cattle and bovine faeces in the literature [96]. Moreover, ST61 has also been implicated in previous raw milk-associated *Campylobacter* outbreak events [34,96]. These findings suggest that the ST61 isolate detected in the present study most likely originated from bovine faecal contamination [95]. Comparative genomic analysis using the in-house database further demonstrated that the ST61 isolate was clonally related (AD = 9) to a human clinical isolate [97]. Overall, our findings suggest that raw milk distributed via milk vending machines may pose a source of human *Campylobacter* infection.

*Y. enterocolitica* was quantitatively detected in 17 samples (13.7%), with bacterial counts ranging from 1.0 × 10^1^ to 1.9 × 10^6^ CFU/mL. When combining quantitative and qualitative approaches, the bacterium was detected in 37 samples (29.8%). All isolates characterised by WGS were assigned to biotype 1A, with one isolate remaining unidentifiable. At the MLST level, four isolates were assigned to sequence type (ST) 3 (11.4%), two isolates were assigned to ST8 (5.7%), seven isolates were assigned to ST118 (20.0%), and one isolate was assigned to ST157 (2.9%).

The gastrointestinal tract of pigs is considered to be the primary reservoir of *Y. enterocolitica* [98]. However, this pathogen has also been detected in the faeces of other animal species, including cattle and sheep [99]. Outbreaks have predominantly been associated with pork and pork products [100], although cases linked to pasteurised milk [101], raw milk products [102], and plant-based foods have also been reported [103,104].

Six biotypes (BT) of *Y. enterocolitica* are distinguished [105,106]. Among these, biotype 1B is regarded as highly pathogenic [107], biotypes 2–5 are moderately pathogenic [108], and biotype 1A has traditionally been classified as non-pathogenic [106,109,110]. However, pathogenicity is not only influenced by biotype, but also by serotype and associated virulence factors (e.g., adhesins and enterotoxin genes), as well as environmental conditions and host-related factors [108,111,112,113,114,115].

To date, only limited data on the prevalence of *Y. enterocolitica* in raw milk are available. Depending on the detection method applied, prevalence rates in bulk tank milk samples ranging from 1.2% to 7.7% have been reported (7.7% [116], 6.1% [117], 1.2% [118], and 2% [5]). Studies addressing the prevalence, and in particular the quantitative contamination of *Y. enterocolitica* in raw milk vending machines, are currently lacking. The prevalence of *Y. enterocolitica* observed in the present study was substantially higher than that reported for bulk tank milk samples. This finding suggests that raw milk vending machines may either provide favourable conditions for the survival and/or growth of *Y. enterocolitica* or act as a potential reservoir for the pathogen.

Exclusively, biotype 1A isolates were recovered from the raw milk samples analysed. Biotype 1A strains are widespread in the environment and are frequently isolated from animal and human faeces as well as from foods, particularly milk and dairy products [119,120,121]. Although these strains were historically considered apathogenic, there is growing evidence from regular clinical isolation in humans [59,122] that at least a subset of biotype 1A isolates may be associated with gastrointestinal disease in humans, particularly in immunocompromised individuals [104,114,120,122].

All four sequence types identified in the present study (ST3, ST8, ST118, and ST157) were found among the biotype 1A clinical human isolates reported by Stevens et al. (2024) [59]. One isolate from that study was additionally clustered with one of our isolates (TP 142, ST unknown, AD = 0). The sequence types ST3, ST8, and ST157 are also regularly isolated from diverse food matrices [98,123,124,125].

In the quantitative analyses, very high bacterial counts of *Y. enterocolitica* (>10^6^ CFU/mL) were detected in some samples. These levels could potentially fall within the range of the minimal infectious dose, which is estimated to be high, approximately 10^6^–10^9^ CFU, depending on the biotype and serovar, and associated virulence factors [126,127].

*L. monocytogenes* was qualitatively isolated from three samples (2.4%). The isolates were assigned to sequence types ST37, ST226, and ST451. *L. monocytogenes* may enter raw milk through various routes, including intramammary infections [128], but it mainly does so via faecal or environmental contamination during the milking process [129], as well as persistent contamination sources such as biofilms in milking equipment [130]. In the context of raw milk vending machines, the ability of *Listeria* spp. to grow under psychrotrophic conditions and form biofilms is of particular relevance [66,130,131,132]. Although the overall risk of listeriosis associated with raw milk consumption is considered low for healthy individuals [9,94], *L. monocytogenes* warrants particular attention due to its potential for severe clinical outcomes [133]. In previous studies conducted in Switzerland, *L. monocytogenes* was not detected in either raw milk or raw milk cheese [26,134]. In contrast, studies investigating raw milk vending machines in Italy reported prevalence rates ranging from 0 to 1.6% [14,135,136].

All three sequence types identified in this study (ST37, ST226, and ST451) are well documented in environmental reservoirs, including surface waters [137,138], and have also been detected in human clinical samples [138]. In particular, ST37 and ST451 are among the most prevalent sequence types associated with cases of human listeriosis in the European Union. According to the EFSA zoonoses report from 2022, ST37 and ST451 were ranked as the third most frequently identified sequence types among clinical *L. monocytogenes* isolates [139]. In 2023, ST37 was further reported as the second most common sequence detected in human clinical samples [140]. Moreover, an ST37 strain was implicated in a listeriosis outbreak in Denmark in 2022 [141]. With regard to ST226, evidence suggests a close association with the dairy production environment [138].

*Salmonella* spp. was not detected in the raw milk samples analysed in the present study, which is consistent with the findings reported by a Swiss study in 2018 [26]. In general, *Salmonella* spp. may enter raw milk through faecal contamination during the milking process or, more rarely, as a consequence of Salmonella-associated mastitis [142,143]. However, given that *Salmonella* spp. are only infrequently detected in faecal samples from Swiss cattle (the prevalence of asymptomatic carriers is approximately 2% [142]), the risk of contamination of raw milk and raw milk products in Switzerland is considered to be very low [90]. Large-scale investigations in Italy (*n* = 60,907) reported a prevalence of 0.03% in raw milk [14], whereas smaller regional studies from the Piedmont region (*n* = 618) [135] and Liguria (*n* = 355) [136] reported prevalence rates of approximately 0.3%. In contrast, studies from the United States identified substantially higher prevalence rates, with pronounced regional differences ranging from 2% to 6.2% (a mean of 3.6%; *n* = 15,318) [2]. These findings are consistent with the generally higher prevalence of *Salmonella* spp. in the gastrointestinal tract of cattle in the United States compared with most European countries [144]. In summary, the available evidence suggests that the risk of Salmonella infection associated with the consumption of raw milk in Switzerland is considered low, but not negligible.

STEC were detected by PCR in four raw milk samples (3.2%). In contrast to the present findings, a Swiss study conducted previously did not detect *stx* genes in any of its samples (*n* = 74) [26]. In an international context, the prevalence of STEC in bulk tank milk or raw milk was reported to be 2.7% in Finland (*n* = 183) [116], 4.3% in the USA (*n* = 15,318) [2], and 4.7% in the European Union (*n* = 443) [94]. The EFSA reported an increase in the prevalence of tested raw milk samples from 1.8% in 2023 (*n* = 567) to 4.7% in 2024 (*n* = 443) [94]. The particular public health relevance of STEC is attributable to two factors: the low infectious dose of approximately 10–100 cells [145] and the potential severity of disease outcomes following infection [146]. Numerous disease outbreak events, caused by STEC in connection with raw milk/raw milk products, have been documented throughout Europe [147,148,149].

In terms of antibiotic-resistant bacteria, raw milk samples were tested for methicillin-resistant *S. aureus* and ESBL-producing Enterobacterales.

Methicillin-resistant *S. aureus* (MRSA) was detected in a single sample (0.8%). The isolate carried *mecA* as the molecular basis of resistance. In Switzerland, only limited data are available on the prevalence of MRSA in raw milk. Huber et al. did not detect MRSA in either 100 bulk tank milk samples or 200 raw milk cheeses in 2009 [150]. Similarly, Zulauf et al. reported no MRSA detection in 73 raw milk samples in 2018 [26]. To date, MRSA has only been isolated sporadically from mastitis samples in Switzerland (2/142; 1.4%) [150]. At the European level, the prevalence of MRSA in raw milk has been estimated at 2.9% (95% CI: 1.3–5.2%) based on meta-analytical data [73], indicating that MRSA occurs more frequently in raw milk in other European countries than in Switzerland. The MRSA isolate identified in the present study belonged to sequence type ST398 (clonal complex CC398), which is typically associated with livestock [151,152,153]. Isolates of ST398 (CC398) have frequently been linked to methicillin resistance in previous studies, with *mecA* detected in 58% of CC398 clones isolated from mastitis cases [78]. In classical MRSA, the methicillin resistance determinant *mecA* is invariably located within the staphylococcal cassette chromosome (*SCCmec*) [154].

One ESBL-producing *E. coli* isolate (0.8%) was recovered in the present study. The isolate belonged to sequence type ST16559 according to the Warwick/Achtman MLST scheme and harboured the ESBL resistance gene *bla*_CTX-M-14_. The ESBL gene *bla*_CTX-M-14_ is frequently reported in association with livestock production [155]. Together with *bla*_CTX-M-15_, it represents one of the most prevalent ESBL determinants worldwide [156]. To date, there is no published evidence of ESBL-producing Enterobacteriaceae isolated from raw milk in Switzerland; only sporadic detections have been reported from mastitis milk [26,157]. This contrasts markedly with findings from Germany, where a study including 866 bulk tank milk samples revealed a 9.5% prevalence of ESBL-producing Enterobacteriaceae. The most frequently isolated species were *E. coli* (75.6%), *Citrobacter* spp. (9.6%) and *Enterobacter cloacae* (6.1%), with the majority of isolates carrying CTX-M-1 group β-lactamases (CTX-M-1, CTX-M-15, CTX-M-14) [158].

Several methodological limitations should be considered when interpreting the results of this study. Temperatures of raw milk samples were not continuously monitored during transport and storage; thus, potential temperature fluctuations may have influenced bacterial growth. Furthermore, detailed information on the technical design of the milk vending machines and the storage duration of raw milk prior to sampling was not recorded, limiting the ability to identify potential sources of contamination and conditions favouring bacterial proliferation. Sampling was restricted to the summer and autumn months, precluding the assessment of seasonal variations in pathogen prevalence.

## 5. Conclusions

The present study provides a deepened insight into the microbiology and safety of raw milk sold through raw milk vending machines in Switzerland. The pronounced variability in total viable counts observed in raw milk samples, as reported in previous studies, indicates substantial differences in the management of raw milk vending machines. The identified hygienic shortcomings underline the need for clearly defined requirements for milk replacement intervals as well as cleaning and maintenance procedures for the vending machines. Operators should be required to implement mandatory self-monitoring of the microbiological quality of milk dispensed from these vending machines.

The detection of *Campylobacter jejuni*, STEC, and *L. monocytogenes* in raw milk samples confirms the exposure risk to foodborne pathogens associated with the consumption of untreated raw milk from these vending machines. The results regarding *Y. enterocolitica* further emphasise its relevance in raw milk and demonstrate that potentially hazardous proliferation may occur even during refrigerated storage. The detection of antibiotic-resistant bacteria additionally highlights the relevance of raw milk safety within a One Health framework. Overall, the findings indicate that numerous risks are associated with raw milk consumption, which can be effectively avoided through appropriate processing, including pasteurisation in particular.

## Figures and Tables

**Table 1:** Predominant bacterial species in samples with TVC ≥ 8 × 10^4^ CFU/mL.

*Pseudomonas* spp.	*Acinetobacter* spp.	*Lactococcus* spp.	*Staphylococcus* spp.	*Yersinia* spp.	Other Genera ^1^
*Pseudomonas fragi* (6 *)	*Acinetobacter* *johnsonii* (3)	*Lactococcus* *lactis* (2)	*Staphylococcus xylosus* (1)	*Yersinia* *enterocolitica* (1)	*Carnobacterium* *maltaromaticum* (2)
*Pseudomonas* *lundensis* (3)	*Acinetobacter* *guilloiae* (1)	*Lactococcus* *raffinolactis* (1)	*Staphylococcus hominis* (1)		*Enterobacter* *hormaechei* (1)
*Pseudomonas* *brenneri* (1)	*Acinetobacter* *albensis* (1)				*Rahnella inusitata* (1)
*Pseudomonas* *corrugate* (1)					*Raoultella* *terrigena* (1)
*Pseudomonas* *antarctica* (1)					*Erwinia* *persicina* (1)
*Pseudomonas* *libanensis* (1)					*Hafnia alvei* (1)
*Pseudomonas* *taetrolens* (1)					*Comamonas* *testosteroni* (1)

^1^ Bacterial species not belonging to the listed genera. * Number of isolates.

**Table 2:** Compilation of all *S. aureus* isolates with their corresponding sample ID, bacterial count, associated sequence type (ST), and harboured enterotoxin genes (SE).

Sample ID	Bacterial Count (CFU/mL)	ST	Enterotoxin Genes (SE)
TP 150	8 × 10^1^	8	
TP 151	1 × 10^2^	8	
TP 159	3 × 10^1^	97	
TP 95	1.3 × 10^2^	151	
TP 98	3 × 10^1^	151	
TP 2	3 × 10^1^	352	
TP 5	1 × 10^1^	352	
TP 28	3 × 10^1^	352	
TP 87	3 × 10^1^	352	
TP 89	4 × 10^1^	352	
TP 100	2 × 10^1^	352	
TP 56	2.2 × 10^2^	389	
TP 59	5 × 10^1^	504	
TP 106	1 × 10^1^	504	*sec*, *sell*
TP 137	9 × 10^1^	582	

**Table 3:** Compilation of all *Y. enterocolitica* isolates with their corresponding sample ID, bacterial count, biotype (BT), and sequence type (ST).

Sample ID	Bacterial Count (CFU/mL) (If Quantitative Detected)	BT	ST
TP 1	-	1A	n/d *
TP 3	1.0 × 10^3^	1A	3
TP 5	-	1A	118
TP 7	-	1A	n/d
TP 8	1.3 × 10^3^	1A	n/d
TP 11	1.0 × 10^1^	1A	n/d
TP 12	-	1A	n/d
TP 14	-	1A	n/d
TP 22	-	1A	n/d
TP 27	-	1A	n/d
TP 29	4.7 × 10^2^	1A	n/d
TP 45	-	1A	n/d
TP 57	1.1 × 10^5^	1A	118
TP 59	-	1A	118
TP 63	-	1A	8
TP 69	2.2 × 10^2^	1A	8
TP 73	-	-	-
TP 77	9.2 × 10^4^	-	-
TP 93	-	1A	3
TP 101	1.4 × 10^2^	1A	n/d
TP 104	2.3 × 10^4^	1A	118
TP 106	-	1A	118
TP 111	-	n/d	n/d
TP 113	6.4 × 10^4^	1A	157
TP 116	-	1A	118
TP 118	-	1A	n/d
TP 123	3.2 × 10^2^	1A	118
TP 125	1.9 × 10^6^	1A	3
TP 126	-	1A	n/d
TP 127	2.0 × 10^5^	1A	n/d
TP 138	-	1A	3
TP 142	2.2 × 10^3^	1A	n/d
TP 149	4.0 × 10^2^	1A	n/d
TP 152	1.0 × 10^3^	1A	n/d
TP 154	-	1A	n/d
TP 156	9.0 × 10^3^	1A	n/d
TP 161	-	1A	n/d

* n/d: no ST number has been assigned in the database yet.

## Data Availability

The WGS data has been deposited at GenBank/ENA/DDBJ under the following accession numbers: *S. aureus:* JBUXLI000000000, JBUXLK000000000, JBUXLL000000000, JBUXLM000000000, JBUXLO000000000, JBUXLQ000000000, JBUXLR000000000, JBUXLS000000000, JBUXLU000000000, JBUXLV000000000, JBUXLX000000000, JBUXLY000000000, JBUXLZ000000000, JBUXMB000000000, JBUXMD000000000; *Listeria monocytogenes*: JBUXLJ000000000, JBUXLN000000000, JBUXLW000000000; *Campylobacter:* JBUXLP000000000, JBUXMC000000000; ESBL *E. coli* TP21: JBUXLT000000000; MRSA TP124: JBUXMA000000000; *Yersinia enterocolitica*: BioProject number PRJNA1359616.

## Abbreviations

The following abbreviations are used in this manuscript:

CFUs	Colony-forming units
EFSA	European Food Safety Authority
ESBL	Extended-spectrum beta-lactamase
HPAI	Highly pathogenic avian influenza
MALDI-TOF	Matrix-assisted laser desorption/ionisation time-of-flight
MLST	Multilocus sequence typing
MRSA	Methicillin-resistant *Staphylococcus aureus*
PCR	Polymerase chain reaction
STEC	Shiga toxin-producing *E. coli*
TBE	Tick-borne encephalitis
TVC	Total viable count
WGS	Whole-genome sequencing
